# SWIMRT: A graphical user interface using the sliding window algorithm to construct a fluence map machine file

**DOI:** 10.1120/jacmp.v7i2.2231

**Published:** 2006-05-25

**Authors:** James C.L. Chow, Grigor N. Grigorov, Nuri Yazdani

**Affiliations:** ^1^ Radiation Medicine Program and Department of Radiation Oncology Princess Margaret Hospital University Health Network University of Toronto 610 University Avenue Toronto M5G 2M9; ^2^ Department of Physics University of Waterloo 200 University Avenue West Waterloo N2L 3G1; ^3^ Medical Physics Department Grand River Regional Cancer Center Grand River Hospital P.O. Box 9056, 835 King Street West Kitchener Ontario Canada N2G 1G3

**Keywords:** IMRT, sliding window algorithm, MLC, computer programming, fluence map

## Abstract

A custom‐made computer program, SWIMRT, to construct “multileaf collimator (MLC) machine” file for intensity‐modulated radiotherapy (IMRT) fluence maps was developed using MATLAB® and the sliding window algorithm. The user can either import a fluence map with a graphical file format created by an external treatment‐planning system such as ${\text{Pinnacle}}^{3}$ or create his or her own fluence map using the matrix editor in the program. Through comprehensive calibrations of the dose and the dimension of the imported fluence field, the user can use associated image‐processing tools such as field resizing and edge trimming to modify the imported map. When the processed fluence map is suitable, a “MLC machine” file is generated for our Varian 21 EX linear accelerator with a 120‐leaf Millennium MLC. This machine file is transferred to the MLC console of the LINAC to control the continuous motions of the leaves during beam irradiation. An IMRT field is then irradiated with the 2D intensity profiles, and the irradiated profiles are compared to the imported or modified fluence map. This program was verified and tested using film dosimetry to address the following uncertainties: (1) the mechanical limitation due to the leaf width and maximum traveling speed, and (2) the dosimetric limitation due to the leaf leakage/transmission and penumbra effect. Because the fluence map can be edited, resized, and processed according to the requirement of a study, SWIMRT is essential in studying and investigating the IMRT technique using the sliding window algorithm. Using this program, future work on the algorithm may include redistributing the time space between segmental fields to enhance the fluence resolution, and readjusting the timing of each leaf during delivery to avoid small fields. Possible clinical utilities and examples for SWIMRT are given in this paper.

PACS numbers: 87.53.Kn, 87.53.St, 87.53.Uv

## I. INTRODUCTION

Intensity‐modulated radiotherapy (IMRT) has become a popular treatment technique for many types of cancer, such as head and neck,^(1)^breast,^(^ ^2^ ^,^ ^3^ ^)^and prostate.^(^ ^4^ ^,^ ^5^ ^)^This technique can produce a conformal dose distribution at the tumor target volume while sparing the normal tissues of the critical organs.^(6)^ IMRT is being used at many centers and may gradually replace some conventional radiotherapy. In IMRT, the modulated fluence distributions or maps are generated by an inverse treatment‐planning system. These fluence maps are converted to a series of individual MLC beam segments and delivered using a dynamic multileaf collimator (MLC) and control software.^(7)^There are basically two approaches to generate the leaf sequence for the required fluence segments: sliding window and step‐and‐shoot. For the sliding window approach,^(^ ^8^ ^–^ ^10^ ^)^ the MLC leaves move continuously while the radiation is on. For the step‐and‐shoot approach,^(^ ^11^ ^–^ ^13^ ^)^ radiation is delivered by individually collimated beam segments in sequence. Although most centers prefer to use the latter delivery approach due to better hardware and software support from the manufacturers, studies on the former approach are still being done.^(^ ^14^ ^–^ ^16^ ^)^ This is because the step‐and‐shoot technique sometimes uses so many small sized segments with associated monitor units (MUs).^(^ ^17^ ^,^ ^18^ ^)^ Moreover, the delivered dose accuracy can be affected by the accuracy of the leaf positioning,^(19)^ the overshoot effect,^(20)^ and dose nonlinearity for small MU delivery.^(21)^ With more and more external treatment‐planning systems and LINAC support of IMRT delivery using the sliding window technique, more studies regarding the dosimetry and mechanical modulation such as the leaf speed are needed.

For the sliding window technique, the fluence map of each IMRT beam calculated by the inverse planning system is converted to a large number of segmental fields with an assigned dose‐fraction order. For a LINAC such as the Varian 21 EX with a 120‐leaf Millennium MLC, an “MLC machine” file can be generated by the planning system and sent to the MLC console of the LINAC. The machine file has all the information about the positions corresponding to the fraction of beam‐on time for the leaves during beam irradiation. To study in detail the mechanical and dosimetric characteristics of the sliding window technique, such as the correlation between the positions of the leaves and the accumulated dose for IMRT, a custom‐made computer program is needed to construct and generate the “MLC machine” file for the LINAC. This program should have the following features:

1. The import of the fluence map should be convenient: fluence maps in the form of popular graphic formats such as jpeg, bmp, and tiff should be recognized by the program. In addition, the user should be able to create or edit a fluence map using the program manually in an Excel® like environment or in the MATLAB® command prompt.
2. A precise and accurate calibration procedure is needed.
3. The software should allow convenient changes or modifications to certain parameters under investigation (leaf speed, dose rate, number of segments, etc.), to apply dose corrections, and to add enhancements to the algorithm.
4. There should be a user‐friendly front‐end graphic user interface (GUI).
5. Simple image‐processing functions such as fluence field edge trimming, region of interest selecting/cropping, and field resizing, should be included in the program.
6. A comprehensive database recording all the fluence map information such as field size, beam energies, MUs, and so on is needed.

A program called SWIMRT was written using MATLAB® and reported in this paper. This program has all the above‐mentioned features and a user‐friendly GUI front end. Only a graphic file of fluence map is needed by SWIMRT to construct and generate an “MLC machine” file, which can be sent to the LINAC to control the MLC for the beam. Radiographic films have been irradiated using the software's “MLC machine” files and have reproduced the fluencies to our expectations. This program has been verified using film dosimetry and is used to study the leaf positional modulation algorithm, leaf leakage/transmission and penumbra effect, and mechanical limitations of the MLC for the sliding window technique.

## II. METHODS AND MATERIALS

### A. MLC and the “MLC machine” file

The Varian 120‐leaf Millennium MLC system contains two carriages, one located directly beneath the X1 jaw and the other directly beneath the X2 jaw. Each carriage holds 60 leaves with 40 inner leaves, each having a width of 0.5 cm at the isocenter. The inner leaves can produce a maximum square field size of $20\times 20\;{\text{cm}}^{2}$. The width of the outer leaves is 1 cm; therefore, all 60 leaves can generate a maximum square field size of $40\times 40\;{\text{cm}}^{2}$. However, for an IMRT beam using the MLC, the leaves travel beyond the central axis with a maximum range of 7.5 cm, making a maximum IMRT field size of $15\times 40\;{\text{cm}}^{2}$. In the Varian 21 EX LINAC, the MLC is positioned as a tertiary system below the adjustable jaws. Each MLC leaf has a rounded end with a linear trajectory, which greatly simplifies the mechanics so that failures should occur less frequently.

For the Varian 21 EX LINAC, the movement of the leaves in the MLC is controlled by the MLC console with an “MLC machine” file.^(22)^ This file controls the position of every leaf in the MLC at each fraction of irradiation dose increment. The file also records the positions of the jaws. It is possible to view, simulate, and edit this file using the Varian SHAPER program (v.6.3 was used in this study). Alternatively, the file can be opened using a word processor such as Microsoft Word as plain text to view and edit the code. In generating the “MLC machine” file, it should be noted that the number of field segments for a single beam is limited to no more than 500. Moreover, the pairs of opposite leaves cannot be positioned less than 0.5 mm away from each other, but they are allowed to be fully closed. This includes the case of one leaf being stationary, and the other closing onto it.

### B. The sliding window algorithm

The sliding window algorithm is well known and is well documented in the literature.^(^ ^23^ ^–^ ^25^ ^)^ In this paper, only the important issues are highlighted. The first task of the algorithm is to use a function to convert a matrix of dose values into a matrix of exposure times; that is, based on the dose rate, the function determines the duration of time that each sample point must be exposed to radiation. The function used is very simple and has an ideal relationship:

(1) $$\text{Exposure}\;\text{Time}=\frac{\text{Dose}}{\text{DoseRate}}.$$

However, a modified function based on Eq. (1) is needed to make convenient corrections to the dose map. For example, to compensate for leakages, one may lower the desired dose at each point using a predetermined equation. To make such corrections, Eq. (1) can be modified to as follows:

(2) $$\text{Exposure}\;\text{Time}=\frac{\text{Dose}-f(\text{Dose})}{\text{DoseRate}},$$

where *f*(Dose) is the dose adjustment for correction. Once the matrix is converted to exposure times, the sliding window algorithm can be applied. The algorithm acts on each leaf pair profile (each row) individually. When the first leaf moves from a certain position, it leaves that position exposed until the second leaf reaches that same point. Two spatially sampled arrays need to be computed by the algorithm, one that contains the times at which the first leaf exposes each point, and the second, which contains the times at which the second leaf covers the point. The first array should always be greater (in time) than the second array, and the difference between the two arrays should be identical to the array of desired exposure times calculated previously. As a starting point, one can assign the first leaf the same values as the array of desired exposure times, and assign the second leaf an array of all zeros. With these assignments, the difference between the two leaf profiles matches the exposure times array as desired. The problem with these arrays is that the second leaf would have to move at an infinite velocity (at time=0, the leaf exists at all positions), and the first leaf would travel backward in time. To fix the time traveling, the portions of the arrays that are at fault can be interchanged between the two leaves, with their profiles inversed as shown in Figs. 1(a) and (b). Figure 1(a) shows an example of a leaf pair exposure‐time profile, and Fig. 1(b) shows that the roles of leaf A and leaf B reversed, making the first half of the profile realizable. After such adjustment as shown in Fig. 1, there is the same problem with leaf A, and vice versa. To solve the time traveling problem, the roles of the leaves can be reversed again, but only from the midposition. The remaining portions of the two profiles are basically being rotated about the line that passes in between the values at which the exchange is occurring, as shown in Fig. 2. This figure shows that the roles of leaf A and leaf B are reversed again, after the midposition. However, the infinite velocity problem still exists. Skewing the profiles according to the maximum velocity of leaves can easily solve this problem. A linear function is added to the profiles:

(3) $$f(d)=\frac{1}{\text{Maximum}\;\text{Velocity}}d$$

where *d* is the position of the leaf. For a maximum leaf velocity of 2.5 cm/s for the MLC used in this study, the profile used in the above example would become as shown in Fig. 3. It can be seen that the profiles for both leaves are skewed to eliminate large velocities. In the example used, the roles were only reversed twice. For more complicated patterns, the arrays may be interchanged many times. The function runs through each leaf profile, from the beginning, exchanging the roles whenever it detects that one of the leaves is time traveling. At this point, how the leaves should behave has been effectively determined in order to deliver the desired fluence. The last step is to generate the “MLC machine” file.

**Figure 1(a):**
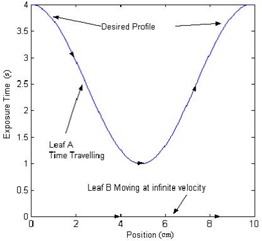
An example of a leaf pair exposure‐time profile

**Figure 1(b):**
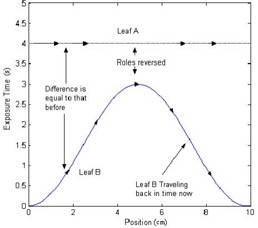
The roles of leaf A and leaf B are reversed, making the first half of the profile realizable.

**Figure 2:**
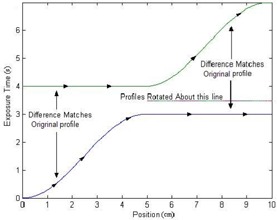
The roles of leaf A and leaf B are reversed again after the mid position.

**Figure 3:**
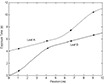
Profiles for both leaves are skewed to eliminate large velocities

To generate the machine file, the arrays must be changed from spatial samples to samples in time. This is accomplished by sampling the array of exposure times at the desired times. The specified number of fields determines the sample times. It is unlikely that the time samples will occur at exact times inside the exposure time matrix, so averaging is used here. For example, the array for the position at 2 s is looked through and has the times 1.95 s and 2.15 s with positions 3 mm and 4 mm, respectively. The position of the leaf at time 2 s will be taken as follows:

(4) $$\begin{array}{rl} d & =\text{startDist}+(\text{endDist}-\text{startDist})\frac{\text{value}-\text{startTime}}{\text{endTime}-\text{startTime}}\\ & =3+(4-3)\left(\frac{2-1.95}{2.15-1.95}\right)\\ & =3.25\;\text{mm} \end{array}$$

That is, if the user wants position *d* at time “value,” a search through the array looking for the time “value” is performed. Usually, the expected exact value is not there, but only the array elements before and after it was found, which are startTime (before) and endTime (after) in Eq. (4). These array elements have distance values of startDist (before) and endDist (after). Before the data can be written to the file, it should be noted that, if, for example, one of the leaf profiles is zero along the entire path, the procedure used above will tell the leaves to scan across the screen while remaining closed. One of the restrictions of the MLC is that a pair of leaves cannot move within 0.5 mm of each other. To ensure that this requirement is met, the function searches through each leaf profile. If a zero profile along the entire profile is found, that leaf pair is set to be closed in the center of the pattern. The function also searches for profiles where the dose is zero for a while at the beginning and/or in the end. If a leaf pair that fits these criteria is found, the leaves are set, at the beginning and/or end to stop and close rather than scan across closed. Once all the motions of each leaf pair have been determined, the MLC information is written to the “MLC machine” file.

### C. Application of the sliding window algorithm in SWIMRT

#### C.1 MATLAB® platform

Since a fluence map of dose intensity matrix is processed in SWIMRT, and some simple image‐processing routines are added to the program, a software platform that involves the manipulation of large arrays and matrixes should be used to develop SWIMRT. The MATLAB® environment has many powerful tools for manipulating, copying, and performing calculations on large matrixes, making it the *de facto* choice for computations involving large matrixes. MATLAB® also has a powerful GUI development tool called GUIDE, which speeds up the development of the user interface window. All functions for SWIMRT were written in MATLAB® version 6.5, and tested in MATLAB® versions 6.5 and 7.1. The GUI was written using GUIDE in MATLAB® version 6.5 and 7.1. Only standard toolboxes were used.

#### C.2 Inputs for the functions in SWIMRT

The main computations of SWIMRT are contained in the function “mlcIMRT.” It requires the following inputs: (1) Dose Matrix, (2) Dose Rate, (3) Start Position, (4) Space Precision, (5) Number of Fields, (6) Leaf Maximum Velocity, and (7) Additional file information.

Dose Matrix is a matrix that contains each leaf pair's dose profile. The matrix is required to have exactly 60 rows because there are exactly 60 leaf pairs. The number of columns is not restricted. The columns represent the spatial sampling of the dose profile, in constant intervals. The top left matrix element (1, 1) corresponds to the Y1‐X2 corner of the collimator, and the bottom right element (60, N), corresponds to the X1‐Y2 corner of the collimator. Dose Rate is simply the dose rate that is going to be used for the treatment. Start Position is the position, in centimeters, to the left of the isocenter where the dose map begins, that is, the position of the first column samples of the dose matrix. Space Precision is the distance, in centimeters/pixel, between samples in the rows of the dose matrix. For example, if the matrix is 60×1000, and the pattern is 10 cm in the X‐jaw direction, space precision would be 0.01 cm/pixel. Number of Fields is the number of fields desired in the “MLC machine” file; this can also be thought of as the time ‐ precision of the machine file. Leaf Maximum Velocity is the maximum velocity of the leaves, which can be determined when commissioning the MLC for the LINAC. Additional file information is the information needed to create the file, such as filename, pathname, patient's name and ID number (if applicable), collimator rotation, and so on.

### D. Verification and implementation

The IMRT beam associated with the MLC leaf movement controlled by the “MLC machine” file was verified. The field intensity matrix of the beam was recorded by our film dosimetric system. Kodak XV and EDR films were used in this paper and were calibrated with 6‐MV and 15‐MV photon beams produced by our Varian 21 EX LINAC. The calibration was done by placing film at the isocenter (source‐to‐axis distance (SAD)=100cm) with 5‐cm solid water slabs $\left(30\times 30\;{\text{cm}}^{2}\right)$ on top. In this setup, the film was positioned at the absolute dose calibration point, calibrated accurately to give 1±2%cGy per MU with a $10\times 10\;{\text{cm}}^{2}$ field using our local standard Capintec PR06C Farmer ionization chamber and Capintec 192 electrometer. The beam intensity map from SWIMRT was measured using film with the same setup as calibration. When the film was exposed, it was developed in the film processor and analyzed using the Vidar VX‐16 densitometer with RIT 113 radiation therapy dosimetry software, version 4. The sensitometric curve fitting was done by the piecewise polynomial routine in the software. The whole irradiated image or selected profiles along the *x*‐ and *y*‐axes can be plotted to compare with the original fluence map imported to the program.

## III. DESCRIPTIONS AND FEATURES OF SWIMRT

### A. Fluence map loading, creating, and editing

**Figure 4:**
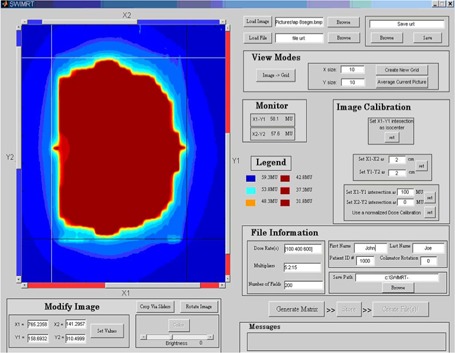
The front‐end window of SWIMRT

**Figure 5:**
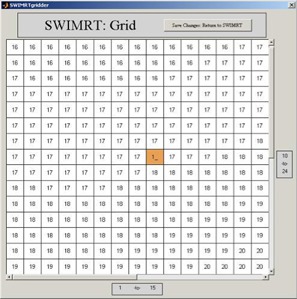
The image‐editable image grid under the subroutine “SWIMRTgridder”

Figure 4 shows the front‐end window of SWIMRT. The fluence map or picture can be loaded or created using the “buttons and boxes” at the right‐hand corner of the window. To create a user‐defined fluence map, the “Create New Grid” button inside the “View Mode” window can be used to generate a grid of the specified size as entered to the “X size” and “Y size” box. The “Average Current Picture” button averages the current map into the specified size to the left, and displays the results in an editable grid. The “Image −>Grid” button is to generate an editable matrix for the imported map or picture. Figure 5 shows the editable matrix in the associated matrix editor “SWIMRTgridder,” in which every element can be modified and changed.

When the editing on the fluence map or picture is finished, the “Save Changes: Return to SWIMRT” button can be pressed. In addition, the map image can be rotated counterclockwise using the “Rotate Image” button at the lower left‐hand corner in the front‐end window (Fig. 4). The color view can be changed using the “Color” button. The brightness of the image can be adjusted using the brightness slider.

### B. Map image cropping and calibration

Map image cropping is accomplished by using the four crop lines (two red and two blue) in the image window in the left of Fig. 4. The four lines are labeled X1, X2, Y1, and Y2, and they can be moved using their individual sliders. Their exact value can be set using the “Set Values” button. It should be noted that the value of the position of the slider is the pixel, or matrix element number. So if a 350pixel×350pixel image is loaded, all four lines will have a minimum value of zero and a maximum of 349. To crop an image for the region of interest, the “Crop Via Sliders” button can be used. When cropping, the pixels along the lines are not cropped but are included in the cropped image.

When the imported map image is modified and the user is satisfied, the position of the isocenter, dose, and image field dimension should be calibrated. The isocenter can be defined simply by the positions of line X1 and Y1 such that their intersection is at the isocenter point. When this is done, the “set” button in the top of the “Image Calibration” window can be pressed. For the calibration of the image dimensions, the positions of all four crop lines should be set such that the absolute distances between X1 and X2 and between Y1 and Y2 are known. The values of the distance are then entered to the small boxes called “set X1−X2” and “set Y1−Y2” in the middle of the “Image Calibration” window. The small “set” button can be pressed when everything is done. For the dose calibration, the crop lines are positioned such that the intersections of X1−Y1 and X2−Y2 occur at positions of known dose. In the “Image Calibration” window, filling in the doses corresponding to the intersections and clicking “set” calibrates the dose and updates the legend and monitor. Another option for dose calibration is to set the dose to be normalized to the maximum occurring dose in the image. To set this calibration, simply click the “Use a normalized Dose Calibration” button.

### C. Averaged dose matrix mode: Final touch‐ups

**Figure 6:**
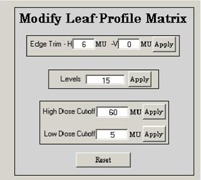
The “Modify Leaf Profile Matrix” window in SWIMRT

When the image calibration is finished, the “Generate Matrix” button at the lower right‐hand corner of Fig. 4 can be pressed. This averages the image over the 5.0 mm of the leaves and applies the dose calibration. When this is done, the “Modify leaf Profile Matrix” window should replace the calibration window as shown in Fig. 6, and the fluence image is replaced by the averaged image. In the figure, there are three small boxes with the following features:

1. Edge trim horizontal transverses the image from both sides, setting any dose smaller than that specified to zero. It stops as soon as it sees a dose value greater than or equal to that specified.
2. Edge trim vertical turns off any leaves that do not make a significant contribution. If any pair of leaves does not have a dose value greater than the specified value, its entire dose profile is set to zero.
3. Levels sets the allowed dose levels, which are linear, from zero to the maximum dose value of the image. For example, if the maximum value is 9 and the levels are set to 10, all the dose values would be set to the closest integer.
4. High dose cutoff sets any dose values above the specified maximum to the specified maximum.
5. Low dose cutoff sets any nonzero dose value below the specified minimum to the specified minimum.

These features are useful for the radiation staff because even if the treatment‐planning system defined the fluence distribution, SWIMRT can carry out the edge trimming on the map. This can help the treatment plan and fluence map QA, especially for the dose coverage on the target with a very sharp or irregular edge. Compared to fluence distributions from the treatment‐planning system, SWIMRT can give other fluence distribution options at the target edge.

### D. Generating and running the “MLC machine” file

When the averaged matrix after all the modifications are satisfied, the “Store” button to the right of the “Generate Matrix” button in the front‐end window (Fig. 4) can be pressed. A “File Information” window at the lower right‐hand corner is filled with appropriate information. The “Multipliers” field allows the user to scale the fluence map by any number of factors. The default value is 1. For the dose rate, number of fields, and multipliers fields, the user can specify more than one value for each, in the form of a vector. For instance, if [100 200 300 400] were entered for dose rates, four files would be created using each dose rate and labeled accordingly. When all information is filled, the “Create File(s)!” button can be pressed. This will start creating the “MLC machine” files. When completed, “finished!” will appear in the message box. However, before loading the machine file to the MLC console, it is good practice to open the file on the Varian SHAPER program to check and simulate the “irradiated” field. If there is no problem with the file, it can be loaded to the MLC console of the LINAC for irradiation.

### E. Importing the “MLC machine” file to SWIMRT

One important feature of SWIMRT is that an “MLC machine” file, whether generated by the treatment‐planning software or SWIMRT using step‐and‐shoot or the sliding window algorithm, can be imported to SWIMRT. The program converts the machine file back to a fluence map shown in the matrix editor, SWIMRTgridder. The user can then modify the map and change the calibration conditions and dose rate according to his or her need. Finally, another “MLC machine” file can be generated using the sliding window algorithm. If the imported machine file from ${\text{Pinnacle}}^{3}$, for example, used the step‐and‐shoot algorithm, SWIMRT can convert this file to use sliding window. This function can benefit the study of comparison between the step‐and‐shoot and the sliding window algorithm using the same MLC. Moreover, such a function can help modify the dose rate, field size, and leaf speed of a created “MLC machine” file. In this study, ${\text{Pinnacle}}^{3}$ version 6.2b was used.

## IV. PROGRAM AND DOSIMETRY VERIFICATION

**Figure 7:**
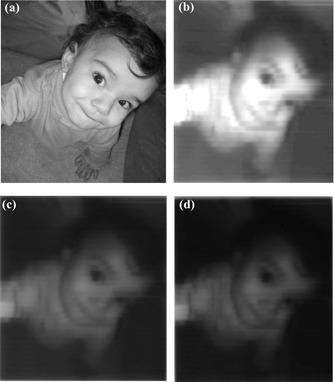
Comparison of the original imported picture to the “copied” IMRT beam images on the films with different irradiation conditions using SWIMRT. (a) An original picture of “baby girl” and beam images converted from (a) using (b) 125 MUs, (c) 175 MUs, and (d) 250 MUs. The films were irradiated at SAD=100cm with 5 cm solid water slab on top using 15‐MV photon beam. The field size of the three beam images is set to $14\times 17.5\;{\text{cm}}^{2}$.

The first verification in this paper is to test the ability of SWIMRT to duplicate a picture on a film irradiated by an IMRT beam using an “MLC machine” file. Figure 7(a) shows the original photo of a “baby girl” used in the verification. This photo, in jpeg format, was imported to SWIMRT. The step‐by‐step procedure was then followed according to Section III, and “MLC machine” files of 125 MUs, 175 MUs, and 250 MUs were generated with field size $14\times 17.5\;{\text{cm}}^{2}$. The files were transferred to the MLC console of the LINAC. Films from the same batch were irradiated with the IMRT beams associated with the machine files and developed. The images of the films for irradiations of 125 MUs, 175 MUs, and 250 MUs are shown in Figs. 7(b), (c), and (d), respectively. It can be seen that the larger the number of MUs used in the irradiation, the darker the images. The intensity of the beam (i.e., MU) can be defined during the calibration procedure of the program. However, due to the limited resolution of the MLC leaves (0.5 cm width along the Y1−Y2 direction, within a field of $20\times 20\;{\text{cm}}^{2}$),^(26)^ a perfect pixel‐by‐pixel duplication for the original picture was not expected.

**Figure 8:**
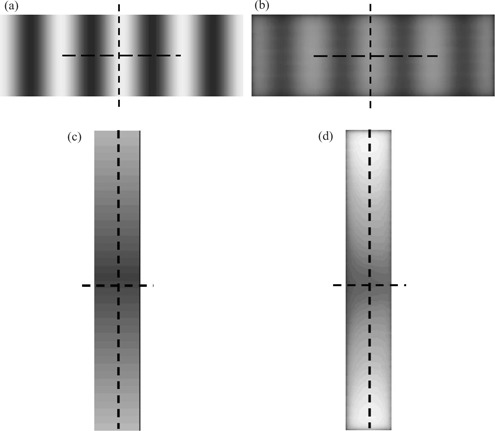
The fluence maps used in the verification of the accuracy of SWIMRT. (a) Original fluence map along the *x*‐axis; (b) irradiated fluence map based on 8(a); (c) original fluence map along the *y*‐axis; (d) irradiated fluence map based on 8(c). The broken lines in the figures represent the horizontal and vertical profiles plotted in Fig. 9.

**Figure 9:**
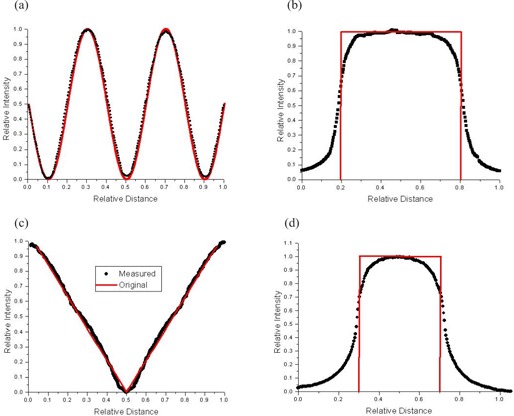
Profiles plotted according to the broken lines in Fig. 8. (a) Horizontal profiles for Figs. 8(a) (line) and 8(b) (dot); (b) vertical profiles for Figs. 8(a) (line) and 8(b) (dot); (c) horizontal profiles for Figs. 8(c) (line) and 8(d) (dot); (d) vertical profiles for Figs. 8(c) (line) and 8(d) (dot). The profiles of Figs. 8(a) and (c) are normalized to the maximum intensity value, while those of Figs. 8(b) and 8(d) are normalized to the irradiated MUs delivered to the isocenter.

To investigate the accuracy of the program in greater detail, some specifically designed fluence maps were created and tested. The beam profiles of the original maps imported from the Varian SHAPER program and their irradiated films (Kodak XV), along the *x*‐axis (between X1 and X2 jaw) and the *y*‐axis (between the Y1 and Y2), were plotted and compared. Figure 8 shows some of the fluence maps used in the verification. Figures 8(a) and (c) are the original fluence maps imported to the program. For Fig. 8(a), the irradiated beam intensity was dominantly modulated by the MLC leaves moving in parallel with the *x*‐axis. For Fig. 8(c), since the beam intensity is varied along the *y*‐axis, the movements of the leaves are perpendicular to the intensity variation. Figures 8(b) and (d) are the irradiated fluence maps generated by SWIMRT and recorded on the films based on Figs. 8(a) and (c), respectively. For Fig. 8(b), the field size was $10\times 3.5\;{\text{cm}}^{2}$. Two hundred and sixty‐eight monitor units were used for a 15‐MV photon beam with dose rate 100 MU/min. For Fig. 8(d), a field size of $2\times 10\;{\text{cm}}^{2}$ and 31 MUs were used with the same irradiation condition as in Fig. 8(b). The horizontal (*x*‐axis) and vertical (*y*‐axis) profiles (broken lines) in Figs. 8(a) and 8(b) are plotted in Figs. 9(a) and 9(b), respectively. Similarly, the vertical and horizontal profiles of Figs. 8(c) and 8(d) are plotted in Figs. 9(c) and 9(d), respectively. For the original profiles, they are normalized to the maximum intensity value; for the measured profiles, they are normalized to their irradiated MUs at the isocenter (i.e., 268 MUs for the horizontal and 31 MUs for the vertical profiles). In Fig. 9(a), it can be seen that both the original and measured profiles match well. In the measured profile (dotted line), the relative intensities at the relative distances of 0.1, 0.5, and 0.9 are not perfectly at zero compared to those of the original profile. This may be due to the artifacts of the film dosimetry system. Figure 9(b) shows that the original and measured vertical profiles (vertical broken lines in Figs. 8(a) and 8(b)) are quite different. In designing the original fluence map of Fig. 8(a), an absolute sharp penumbra edge was tested with the irradiated field. The larger penumbra of the measured field was formed by the MLC leaves along the *y*‐axis and was well known and unavoidable. Figure 9(c) shows the vertical profiles along the *y*‐axis, and it can be seen that both the original and measured profiles agree well. In Fig. 9(d), which shows the horizontal original and measured profiles, a larger penumbra of the measured profile can be found as in Fig. 9(b). In Fig. 9, it can be seen that due to the penumbra effect and finite width of the leaf, it is difficult to completely duplicate a perfect fluence profile along the *y*‐axis. This is a general mechanical limitation of the MLC.

**Figure 10:**
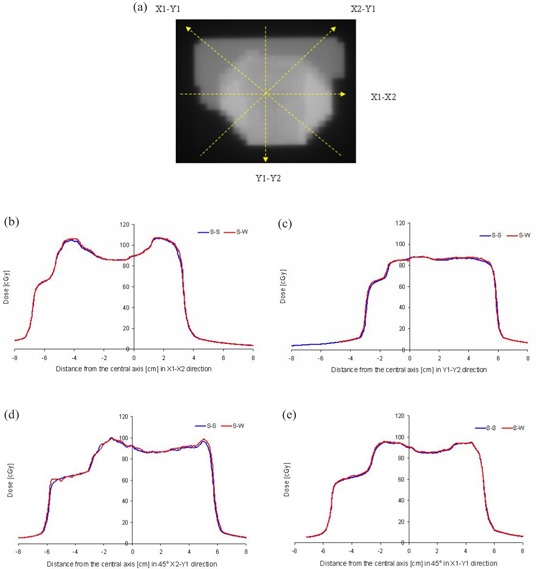
(a). Fluence map for an anterior‐posterior segmental beam for the prostate IMRT. The broken lines represent the beam profiles measured at the (b) X1‐X2, (c) Y1‐Y2, (d) 45° X2‐Y1, and (e) 45° X1‐Y1 directions. The blue curve is the profile using the step‐and‐shoot algorithm (S‐S) from Pinnacle; the red curve is the profile using the sliding window (SW) algorithm from SWIMRT.

For the verification of a clinical fluence map, a prostate patient with staged 2C prostate cancer was treated with IMRT using five coplanar 6‐MV photon beams of equal angular separation of 72°. Figure 10(a) shows an original fluence map of an anterior‐posterior segmental beam. This fluence map was exposed on Kodak EDR film using “MLC machine” files generated by the step‐and‐shoot algorithm of ${\text{Pinnacle}}^{3}$ version 6.2b and SWIMRT's sliding window algorithm. It should be noted that this version of ${\text{Pinnacle}}^{3}$ does not support the sliding window algorithm. Films were placed inside a solid water phantom at SAD=100cm with 11 cm solid water slab on top. The X and Y jaws were open to $8\times 8\;{\text{cm}}^{2}$, and a dose rate of 400 MU/min was used. Figures 10(b) to (e) show the beam profiles measured according to the broken lines in Fig. 10(a). It can be seen that the profiles generated by the step‐and‐shoot (blue curves) and the sliding window (red curves) algorithm match well with each other based on the same fluence map from ${\text{Pinnacle}}^{3}$. The small deviation (<5%) between the step‐and‐shoot and the sliding window profiles in the figures may be due to the uncertainty of the film dosimetry and the mechanical instability of the MLC leaf movement using the sliding window algorithm.

## V. DISCUSSION AND FUTURE WORK

For the dosimetry, the method used in the program for calibration is to relate the pixel values to dose values. The user enters two points and specifies the dose values desired for them. SWIMRT uses a linear fit through the two points to extrapolate dose values for other pixel values. If the calibration causes some pixel values to have a negative dose, they will be set to zero dose. It would be fairly straightforward to use a nonlinear dose calibration, such as a spline fit through any number of user‐specified points, or a piecewise continuous linear fit through an unlimited amount of user‐defined points. This would increase the program's flexibility for the user and will be implemented in SWIMRT in the near future.

Although the original aim to develop SWIMRT is to improve the sliding window IMRT delivery, clinical implementation of SWIMRT is possible. For example, it is possible to adjust/modify the unwanted hot/cold spot in the fluence map for an IMRT beam using SWIMRT instead of handling it in the treatment‐planning system.^(27)^ With the SWIMRT fluence map editor, physicists and dosimetrists can edit the fluence map generated by the treatment‐planning system inversely, and then obtain the “MLC machine” file directly for QA purposes. The radiation staff working in the plan evaluation preferred this fluence map editing feature. In addition, physicists can use SWIMRT to design and edit their specific fluence maps for the routine MLC machine QA.

**Figure 11:**
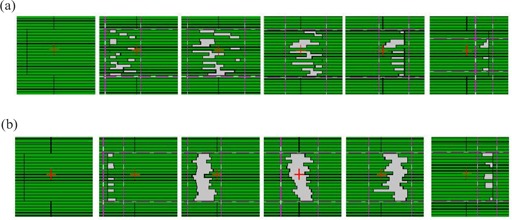
(a) An example of a sliding window irradiation with time going from left to right. It can be seen that some leaves scan across very fast, while some move slowly. This results in many small subfield and penumbra errors. (b) The same example in (a), but the faster leaves’ “takeoff” time is delayed to maximize the grouping of leaves, which minimizes the small field and penumbra errors.

For the present sliding window algorithm used in SWIMRT, more accuracy can be achieved by considering the latency effect of the MLC leaves.^(^ ^28^ ^,^ ^29^ ^)^ This correction can be determined by measuring the actual leaf opening time versus the programmed opening time. Moreover, the dosimetric impact of the tongue‐and‐groove effect should be considered.^(30)^ For future software updates, we are going to focus on the problems of the field distribution within the beam and adjusting the timing of each leaf in the delivery. Regarding the former problem, SWIMRT uses evenly spaced segmental fields, or samples, of the leaves’ movements within a single IMRT beam. However, in some fluence maps, the pattern may change a lot more in certain areas than in others. It would be interesting to see how the accuracy of the beam delivery would increase by assigning a nonlinear sampling array, one that has more samples in the area where the pattern changes more rapidly. Regarding the latter problem, many studies have shown that the small field and leaf penumbra effects reduce the accuracy of dose delivery.^(^ ^31^ ^–^ ^33^ ^)^ For most patterns, the leaves move at different rates, producing errors from small individual fields and penumbras by the adjacent leaf pairs. Figure 11(a) shows an example of a sliding window irradiation. It can be seen that some of the leaves can cross very fast, and some move slowly. This leads to a lot of small individual fields and penumbra errors. It may be possible in some cases to reduce these errors by adjusting the timing of each leaf, so that they move in unison with the other leaves as much as possible, as shown in Fig. 11(b). In the figure, it can be seen that the “takeoff” time of the faster leaves is delayed, in order to maximize the grouping of the leaves and minimize the penumbra and small field errors. With the “building‐block” programming structure used in SWIMRT, the user can modify and change the sliding window algorithm to reduce the delivery time and avoid the small fields. The above works are in progress.

## VI. CONCLUSION

An “MLC machine” file generation program, SWIMRT, was developed based on the sliding window algorithm. The program was written using MATLAB® and has a user‐friendly front‐end window. The user only needs to import a fluence map as a graphic file to the program, and an “MLC machine” file can be generated. The fluence map can either be calculated by a treatment‐planning system or designed by the user. Comprehensive calibrations for the dose and field dimension of the imported map are needed before generating the machine file. The user can resize the field or trim the field edge. Moreover, the imported fluence map matrix is editable so that the beam intensity of the “irradiated” IMRT beam can be readjusted. When the edited map is not appreciated by the user, the program allows him or her to return to the previous status for further correction. The generated machine file is adaptable to the Varian SHAPER program and can be sent to the MLC console of the LINAC. The IMRT beam modulated by the machine file can then irradiate a field in the same way as the imported fluence map with the specified photon beam energy. The program was verified by comparison between the original fluence maps and their irradiated beams measured on films. It is understood that perfect duplication is not possible due to the limitation of the MLC leaf width for resolution, leaf penumbra effect, leaf leakage/transmission, and uncertainty of the film dosimetry.

This program was developed to study the IMRT sliding window delivery such as optimizing the sliding window algorithm, investigating the leaf leakage/transmission and penumbra effect, and the mechanical limitations of the MLC. Although IMRT planning and delivery can be carried out using the sliding window technique in the newer version of external beam treatment‐planning systems such as ${\text{Pinnacle}}^{3}$ version 7.x, a custom‐made program like SWIMRT is essential in generating and editing custom “MLC machine” files with our one‐of‐a‐kind image processing and calibration features for research and development.

## ACKNOWLEDGMENT

All measurements for this study were done in the Grand River Regional Cancer Center. The software was first developed when JCLC was at GRRCC in 2005. The authors thank Dr. Rob Barnett for his solid support and valuable comments in this study.
